# Implicit Affect and Autonomous Nervous System Reactions: A Review of Research Using the Implicit Positive and Negative Affect Test

**DOI:** 10.3389/fpsyg.2019.01634

**Published:** 2019-07-16

**Authors:** Anna-Sophie Weil, Gina Patricia Hernández, Thomas Suslow, Markus Quirin

**Affiliations:** ^1^Department of Psychosomatic Medicine and Psychotherapy, Leipzig University, Leipzig, Germany; ^2^Stress and Health Research Group, Department of Basic Psychology, Universitat Autònoma de Barcelona, Barcelona, Spain; ^3^School of Management, Technical University of Munich, Munich, Germany; ^4^PFH Göttingen, Göttingen, Germany

**Keywords:** implicit affect, dual-processing model, implicit positive and negative affect test, autonomous nervous system reactions, cardiovascular responses

## Abstract

In everyday life, affective processes occur spontaneously and typically go along with an automatic activation of action tendencies and physiological responses. Because self-reports of affect are also known to be biased by various factors, including deficits in introspection or impression management strategies, an indirect measure, the Implicit Positive and Negative Affect Test (IPANAT), was developed to assess implicit affect and to circumvent these difficulties. In this review, findings from neurobiological and clinical studies administering the IPANAT are revised, we focus on the link between implicit affect and psychophysiological reactions to affective stimuli and stressors. Specifically, implicit affect as measured by the IPANAT was found to predict cardiovascular, endocrine, and functional neuroimaging correlates of stress or fear beyond explicit affect. The present evidence strongly suggests the usage of implicit affect measures in future research on stress and psychopathology.

## Introduction

Affects refer to basic, evolutionary conserved processes (Penner and Stoddard, 2018) as one of their many purposes is to provide information about our situation, including information about whether our goals may be thwarted or attained (Montag and Panksepp, 2017). There is agreement that affects comprise different components such as situation appraisal, subjective feelings, expressive behavior, physiological responses, and action preparation (Scherer and Moors, 2019).

Although the details of affective experience are thought to be molded by individual familiarity with the encountered situation and socialization, the basic affective reactions are most likely evolutionary shaped processes (Damasio, 2002; Mason and Capitanio, 2012). As parts of affect, cognition, physiology, as well as individual and situational differences play an important role in the affective experience. Furthermore, cultural influences (Quigley and Feldman Barrett, 2014) coupled with language and verbal manifestations (Cacioppo et al., 2000) have to be considered when trying to understand affect. In general, we can divide affect into two broader groups of positive and negative valence (i.e., dimensional approach), or we can see it as a rather small number of basic and distinct affects (i.e., categorical approach) (Panksepp and Watt, 2011; Tracy and Randles, 2011). Contemporary appraisal theories define affects as processes rather than states. The basic premise is that affects are adaptive responses that reflect appraisals of features of the environment that are important for the survival of the organism (Moors, 2013). Therefore, the study of the nature of these processes (i.e., if they are conscious or unconscious) is critical for emotion research. Another notion is that appraisal processes may be automatic and conscious at the same time (Lieberman, 2019), a conceptualization where conscious affective experiences are considered to be composed by a pre-reflective (i.e., automatic) and a reflective (i.e., rational) process. In this line of inquiry, the IPANAT (Quirin et al., 2009a) has been developed for the assessment of implicit affect, here conceptualized as the automatic and pre-reflective component of the affective experience.

To shed more light on the physiological components of emotions and how they may relate to the development of psychological and psychosomatic disease, previous research investigated relationships between self-reports of affect and ANS reactivity. However, the literature is inconsistent, which can partly be attributed to the multifunctionality and complexity of ANS processes, but also to introspective limitations concerning different emotional components (e.g., Kreibig, 2010). Because of these introspective limitations, it can be considered informative to investigate the link between automatic (“implicit”) components of affect with ANS reactivity and its potential consequences for health (Brosschot et al., 2014; van der Ploeg et al., 2016). This is particularly the case as automatic affective processes influence behavior over and above affect self-reports (Winkielman et al., 2005; Gainotti, 2012). The present article provides an overview of initial empirical evidence on this link, with most of the reviewed studies capitalizing on the IPANAT to assess implicit affect. We hope to stimulate enhanced usage of implicit measurement in future research on physiological components of affect.

## Implicit Affect and Its Theoretical Background

Behavioral research has for a long time been struggling with measuring important constructs without tapping into problems associated with explicit measurements. Explicit measures or self-reports, directly accessing the constructs in question, deal with problems like social desirability and lack of introspection. To avoid such problems, researchers developed implicit measures. These new approaches used methods like priming, reaction time techniques or word-fragment completion tasks intending to assess and operationalize concepts like attitudes, self-concepts and affect from another angle (Fazio and Olson, 2003).

Often, dual-processing models have been used as a rationale for distinguishing implicit from explicit measurement, which can also be applied to the case of implicit affect. Most appraisal theories of affect are in line with a dual-processing view (e.g., Clore and Ortony, 2000). According to this view, information can be processed with propositions and rules, which convey one or more appraisal values. Alternatively, information can be processed in an associative way, activating learned associations between representations of the stimuli and previously stored appraisal outputs (Moors, 2013).

There is a debate about whether these two types of processes are conscious or unconscious (Moors, 2010). As mentioned above, Lieberman (2019) argues for the possibility that appraisal processes are automatic but also conscious. According to this author’s view, there are at least two types of conscious processes, reflective and pre-reflective, collectively they constitute the basis of our immediate conscious experience, but pre-reflective processes share the computational features of automatic processes. Accordingly, affective experience would initially be a pre-reflective process with several simultaneous automatic processes giving rise to not-yet-reflected upon experience. This automatic connection between perceptual and body response systems constitutes first manifestations of affect.

In line with an approach toward affect as information processing, implicit affect is conceptualized as the automatic activation of cognitive representations of affective experiences (Quirin et al., 2009a). For the authors, implicit affect, albeit not processed on the level of reflective cognition, is nonetheless considered to be based on cognitive representations of affective experiences processed in a pre-conceptual mode rather than a conceptual mode (Quirin et al., 2009a). That is, on the basis of an associative processing system, the stimulation of one representation within the system is spreading in a way that a larger network of representations is activated (Gyurak et al., 2011). Within appraisal theories this associative mechanism is viewed as an automatic activation of learned associations between stimulus representations and previously stored appraisal outputs (Moors, 2013). Where explicit affect (i.e., reflecting on our affective state) is not necessarily congruent to pre-reflective states, and can even alter them (Lieberman, 2019).

The IPANAT relates to this pre-reflective dimension of affect, using affect priming of judgments as a method to assess implicit affect. Specifically, the test draws on the principle of affect infusion, according to which affect exerts an impact on evaluative processes influencing the judgments of unrelated objects. It has been shown that judgments about ambiguous objects such as artificial words require a constructive cognitive process that capitalizes on a vast amount of currently accessible information (Forgas, 1992). The more ambiguous a stimulus is and the less of a predefined meaning it has for an individual, the smaller is the amount of available knowledge directly related to the stimulus, leaving more space for affective states to automatically influence judgments of the stimulus (Bower, 1981). Thus, the goal of the test is to capture the pre-reflective affective process expressed in the participants’ biased judgments via the principle of affect infusion. According to the authors, the fact that participants are not asked to reflect or to give information about their affective states adds to the meaning of implicitness for the test. This is even more so the case as the rates of participants’ suspicion that the IPANAT might measure affect is lower than one percent (Quirin et al., 2009a).

Accordingly, the IPANAT uses participants’ ratings of the degree to which six artificial words (SAFME, VIKES, TUNBA, TALEP, BELNI, and SUKOV) sound like six mood adjectives (happy, cheerful, energetic, helpless, tense, and inhibited). Thus, the test is composed of 36-items, which are scored on a 4-point Likert scale, ranging from *doesn’t fit at all* to *fits very well*. From the responses, a summary over all negative judgments is calculated as an indicator for INA, and over all positive words as an indicator for IPA. Evidence for internal consistency, test-retest reliability, stability, criterion, construct and etiological validity of the IPANAT has been shown (Quirin et al., 2009a,b; Brosschot et al., 2014; Quirin and Bode, 2014; Mossink et al., 2015; van der Ploeg et al., 2016). Recently, proof for cross-cultural validity emerged (Quirin et al., 2018).

We might question how the IPANAT is comparable to other measures of implicit affective processes. For example, the Implicit Association Test (IAT; Greenwald et al., 2003) has been developed to indirectly assess attitudes on the basis of delays versus facilitations in response to key presses that have double meanings. In the case of the IAT-Anxiety (Egloff and Schmukle, 2002) participants are instructed to make a series of category judgments (referring to different pairs of the target categories “self” vs. “other” and “anxiety” vs. “calmness”). Accordingly, the IAT can measure implicit self-concepts (attitude toward oneself) of affect rather than affect immediately. A similar argument can be made for differentiating the measurement of the IPANAT from that of the Affect Misattribution Procedure (AMP; Payne et al., 2005), which measures attitudes (rather than affect immediately) via key response to Chinese ideographs following pictures of the attitude target.

In addition, the IPANAT differs from these two procedures in that it does not treat positive and negative as two poles of one dimension but as two different dimensions, which has been consistently replicated with factor analyses (Quirin et al., 2009a, 2018; Sulejmanov and Spasovski, 2018). This is in line with two-dimensional models of affect (e.g., Watson et al., 1988) and enables to investigate differential relationships of positive versus negative affect with other variables (Quirin et al., 2009a,b, 2011). Therefore, the procedure of the IPANAT itself lends to higher levels of construct validity with respect to measuring affect (instead of attitudes or self-concepts). Still, this leaves open the question of what kind of connections we will find when comparing the implicit measurements empirically.

Finally, there are findings indicating the neurobiological and behavioral relevance of the concept of implicit affect. For example, the construction of affective experiences is discussed as an interplay between and integration of explicit and implicit affect (Quirin et al., 2011; Quirin and Lane, 2012), its value for cognition (Quirin et al., 2011; Kazén et al., 2015), decision making (Tamir et al., 2015), influence on behavior without eliciting explicit affect (Gendolla, 2012; Tamir et al., 2015), and effort-related cardiac responses (Freydefont and Gendolla, 2012). Accordingly, implicit affect constitutes a construct of strong relevance in psychophysiological and clinical research as well as beyond.

## Autonomous Nervous System

The ANS, as a major component of the human nervous system, is working involuntarily and without consciousness. According to many evolutionary theories, affects organize the activity of the ANS (Levenson, 2014). As a “highly sophisticated system of control” (Brindle et al., 2014, p. 113), it regulates in balance with the central nervous system vital bodily functions like respiration, cardiovascular responses, digestion, metabolism, and organ functions as important parts of homeostatic adjustments and various emergency situations (Folkow, 2000). It is partly located in the central and peripheral nervous system, while we distinguish between a sympathetic (performance-enhancing) and a parasympathetic (relaxation-promoting) branch (Cacioppo et al., 2000; Critchley et al., 2000; Quigley and Feldman Barrett, 2014). Its complexity and fine interactions are illustrated through the interplay of hormonal and neuronal pathways (Folkow, 2000) as well as sympathetic and parasympathetic branches (Brindle et al., 2014). That is why we renounce from a detailed description of either its various functions or multifaceted ways of working and concentrate on giving an overview of the most common ways to measure ANS indices and its significance for psychophysiology.

As such, the ANS also influences cardiovascular responses, is connected to the system of heart and blood vessels. Heart rate and blood pressure, which are typically applied to assess the functioning of these systems, are multiply determined end points of bodily functions. It is not quite clear how exactly heart rate and blood pressure are modulated but the ANS, sympathetic activation in particular, seems to play a central role. A metabolically driven increase in heart rate and blood pressure can be observed due to physiological demands, but also as a response to psychological stress (Brindle et al., 2014). Heart rate variability is commonly used to investigate autonomic vascular control. While a decrease of heart rate variability power is thought to signal a sympathetic activation, an increase is interpreted as the opposite (Reyes del Paso et al., 2013). Total peripheral resistance is used to measure the resistance of vessels and blood viscosity against the blood stream generated by the heart as an index of vasoconstrictive and elastic properties of the peripheral vasculature. Heightened total peripheral resistance is considered a way to measure sympathetic activity (Hill et al., 2013).

Also widely used for exploring autonomous physiological reactions is cortisol as hormonal end product of the hypothalamus-pituitary-adrenal axis mediating many metabolic processes (e.g., enhancing cardiovascular output, respiration, energy delivery, modulating immune responses) to adapt to environmental challenges. Cortisol is released following a diurnal rhythm, meaning cortisol levels reach short and high peaks, especially during the second half of the night. In addition, there is an increase within 20–30 min after awakening in the morning, called the cortisol awakening response. The exact function of the cortisol awakening response remains an open question, but it is discussed as part of anticipation processes regarding upcoming demands of the day (Fries et al., 2009).

## Connections Between Implicit Affect and Autonomous Nervous System

According to Cacioppo et al. (1993) the relationship between affect and more broadly investigated physiological reactions is to some degree ambiguous and highly context-dependent (see also Mauss et al., 2005; Bradley and Lang, 2007; Kreibig, 2010). According to Evers et al. (2014) weaknesses of response coherence across multiple affect components might depend on the degree responses take place automatically or involve reflective cognitive processes. Thus, implicit affect as part of a pre-reflective process could shed light on the connection between ANS reactions and different affect components. For example, limbic-system structures such as amygdala, hypothalamus, and the mesolimbic dopaminergic system play a major role in physiological and endocrine components of affect (e.g., Cacioppo et al., 1999). At the same time, cortical and paralimbic areas such as ventromedial prefrontal cortex, ventral and pregenual anterior cingulate cortex, anterior insula, somatosensory cortex, and right parietal cortex play a major role in the generation of implicit affect or “background feelings” (Lane, 2008). Still, distributed network models of the emotional brain emphasize the interplay between a large range of neural systems participating in affective processes and relativize the functional specialization of specific cerebral areas (Pessoa, 2013).

Although implicit affect and physiological reactions to affective stimuli are different components of affective experiences, affect-related physiological responses, cognitive appraisals, subjective feelings, and expressive behavior are considered to activate implicit affect representations, via increments in the accessibility of valence-congruent cognitive representations (Quirin et al., 2009a). In fact, there is first evidence that implicit affect is related to affective-physiological experiences.

According to Suslow et al. (2015) INA as measured by the IPANAT is associated with activation of subcortical, striatal areas in response to briefly shown threatening body language and its recognition, demonstrating the potential usefulness of implicit affect measures in predicting spontaneous brain reactions to affective stimuli. In addition, and in line with the theory that unconscious (prolonged) stress plays an important role within the etiology of psychiatric diseases (Davis et al., 2017), there is evidence for a connection between implicit affect and automatic psychophysiological stress responses.

For example, Quirin et al. (2009b) found that INA but not explicit negative affect and IPA predicted cortisol responses to immediate noise threats. By contrast, IPA was reversely related to the cortisol awakening response but unrelated to INA. Compatibly, Mossink et al. (2015) observed an indirect connection between INA, IPA, and cortisol levels in a 24-h ambulatory study, while explicit affect had no predictive power. Implicit sadness was positively related to next day’s cortisol awakening response, indicating that implicit sadness could play a role in the anticipation reactions of next day’s stressors. In sum, the latter findings indicate that implicit compared to explicit affect has a higher predictive value for processes of cortisol secretion and regulation.

Another avenue of research focused on the associations between implicit affect and cardiovascular stress response and recovery. Specifically, Brosschot et al. (2014) found that blood pressure recovery after harassment induction was faster for individuals high in implicit positive or low in INA. van der Ploeg et al. (2016) demonstrated that high INA was related to higher systolic blood pressure, lower heart rate variability and total peripheral resistance after harassment, while there was no such linkage for explicit measures. Low IPA was partly related to slower recovery. This is in line with dual process models, which postulate two distinct modes of information processing, implicit versus explicit, operating relatively independent from each other and showing different effects on physiological and behavioral outputs (e.g., Evers et al., 2014). All in all, the above-mentioned findings suggest that low IPA and high INA as assessed by the IPANAT could play independently from each other roles in prolonged cardiovascular stress recovery.

## Limitations and Considerations for Future Studies

To our knowledge, this is the first review focusing on implicit affect (measured by the IPANAT) and physiological processes. Although we have not fully understood the way implicit affect, as measured by the IPANAT, intertwines with explicit affect, ANS functions and other human reactions, it is certain that the outlined findings and models regarding implicit affect point to a promising approach of gaining more knowledge about affect, functions of the ANS and their interplay.

To further specify what kind of information we can obtain from the IPANAT more research is needed to analyze the relations to other implicit measures or linkages to subtle, involuntary facial expressions, for example measured by the Facial Action Coding system (Ekman and Friesen, 1978). The findings for associations between cardiovascular responses and implicit affect are also raising the question whether the connection is due to the state or trait part of implicit affect measured by the IPANAT (Brosschot et al., 2014; van der Ploeg et al., 2016). More studies are needed to provide such insights into the nature of implicit affect.

To further explore links between ANS reactions and implicit affect, we advise to consider different ways of measuring ANS functions. Regarding measurements already used, heart rate variability is thought to be an adequate index of affective responsivity only if the stimuli have strong affective value (Choi et al., 2017) and is also a controversial measure of sympathetic tone (Reyes del Paso et al., 2013). Against this background, it might be valuable to use other kinds of physiological measures. For example, Kreibig (2010) points out the importance of respiratory indices, especially for investigating distinct affects like fear and anger. Skin conductance is also an essential index for affective and physiological reactions (Hill et al., 2013). Moreover, salivary secretion, influenced by sympathetic and parasympathetic nerves (Proctor and Carpenter, 2007), also shows sensitivity to affective reactions in terms of declining when the individual is disgusted (Vicario et al., 2017) or in a tense, concentrated state (Gemba et al., 1996).

In addition, it may be helpful to control characteristics of the task such as time within the day, sex, age, and race. This is because these variables can significantly influence ANS reactions (Fries et al., 2009; Hill et al., 2013; Brindle et al., 2014; Choi et al., 2017) or diminish the accuracy of the IPANAT, e.g., due to repetition (Brosschot et al., 2014) or small manipulation effects (Quirin et al., 2009b). Furthermore, combining the IPANAT with neurotransmitter measurements seems to be promising because there is evidence that neurotransmitter circuits, underlying affective and autonomous nervous functions, are overlapping (Ehlers and Todd, 2017). Another open question remains the kind of role different areas and networks of the brain play in processing implicit affect. For example, it has been argued that cognitive judgments are influenced by concurrent physiological parameters that may be recognized in interoceptive brain areas (e.g., insula; Lane, 2008; Craig, 2009) and integrated with cognitive aspects in the ventromedial prefrontal cortex as an affective-cognitive convergence zone (Lane, 2008), however, these assumptions still need to be tested. In view of the diverse findings and sometimes small effect sizes for linkages between affect measured by the IPANAT as well as explicit affect with ANS reactions, more research should be conducted looking for possible moderator or mediator variables (Cacioppo et al., 2000), for example implicit memory biases (Mossink et al., 2015).

As IPA and INA show different patterns of relations for example with cortisol reactions (Quirin et al., 2009b; Mossink et al., 2015), it is also possible that we can observe complex connection patterns, depending on the valence of implicit affect we measure, the bodily response in question or point of time within the cycle of the physiological reaction. Also there are mixed findings concerning distinct patterns of ANS functions for basic explicit affects (Cacioppo et al., 2000; Kreibig, 2010; Quigley and Feldman Barrett, 2014). Possibly, within this research context a special variant of the IPANAT assessing discrete affects (Quirin and Bode, 2014) could be administered.

The assessment of implicit affect could also provide more insight into the etiology and treatment processes of psychiatric diseases. For example, there is evidence that (long term) both unconscious stress and IPA (as a “stress buffer”) play an important role in stress recovery, and therefore in handling stressful situations (Quirin et al., 2009b; Brosschot, 2010; Brosschot et al., 2014; van der Ploeg et al., 2016). Liu et al. (2018) found hints for dysfunctional implicit affective regulation in individuals with high anxiety. INA predicted a bias for gaze behavior toward sad faces in healthy young individuals without a history of clinical depression (Bodenschatz et al., 2018). Because negative attentional biases are thought to represent important trait-like characteristics of individuals vulnerable to depression (Gotlib and Joormann, 2010), the finding of Bodenschatz et al. (2018) demonstrates the potential utility of implicit affectivity measures in studying depression-relevant cognitive vulnerability. Additionally, dissociations between implicit and explicit affect can also help to gain insights into the functions of affective symptoms. For individuals with borderline personality disorder higher explicit negative affect but similar INA in comparison to healthy individuals was found, adding evidence to the assumption that negative affect in borderline personality disorder can have appellative functions (Dukalski et al., 2017). Malhi et al. (2007) found that euthymic bipolar patients are less responsive to implicit affect induction. Not least, Marwood et al. (2018) suggested using implicit affect measures to validate therapy success, which seems to be a promising approach as supplement of self-report and explicit measures of affect. The value of this approach might be demonstrated by Remmers et al. (2016) showing that mindfulness improves implicit affect regulation.

In summary, the IPANAT as an economic instrument for the assessment of implicit affect has been shown to be associated with ANS functioning such as stress-related cardiovascular activity and cortisol secretion, beyond explicit methods adding another interesting dimension with its own explanatory value. Accordingly, it appears essential to differentiate between implicit and explicit pathways of affective processing. The IPANAT represents a promising research tool that may help to broaden our knowledge about ANS functioning, affective processes and their interplay in both healthy individuals and those with mental disorders.

## Author Contributions

TS, A-SW, and MQ conceived the outline for this review. A-SW wrote the manuscript with revisions and contributions from GPH, TS, and MQ.

## Conflict of Interest Statement

The authors declare that the research was conducted in the absence of any commercial or financial relationships that could be construed as a potential conflict of interest.

## Abbreviations

ANS: autonomous nervous system
INA: implicit negative affect
IPA: implicit positive affect
IPANAT: implicit positive and negative affect test.
